# Determining online consumer’s luxury purchase intention: The influence of antecedent factors and the moderating role of brand awareness, perceived risk, and web atmospherics

**DOI:** 10.1371/journal.pone.0295514

**Published:** 2024-02-23

**Authors:** Muhammad Ussama Majeed, Hira Aftab, Ali Arslan, Zulaikha Shakeel

**Affiliations:** 1 Department of Management Sciences, National University of Modern Languages, Lahore Campus, Lahore, Pakistan; 2 Institute of Business & Information Technology, University of the Punjab, Lahore, Pakistan; 3 National College of Business Administration & Economics, Lahore, Pakistan; HUTECH University, VIET NAM

## Abstract

The Internet has become the fastest-growing way to sell luxury products. Purchase intention for luxury products in online stores has taken attention in the last few years since the sector has proliferated. The primary objective of this study is to examine the impact of various factors such as Product knowledge, Price consciousness, Perceived enjoyment, Perceived ease of usage, and usefulness on online luxury purchase intention in developing countries like Pakistan. Data was collected from 267 luxury fashion customers in Pakistan through an online questionnaire, and the results were analyzed using Smart PLS-SEM. In addition, the paper investigates the moderating effect of Perceived risk, Brand awareness and Web Atmospheric on the link between the Attitude and Online Luxury Purchase Intentions of the consumer to buy luxury fashion products online. The framework of this study is validated by structural equation modelling (SEM). The findings of this study show that perceived enjoyment, price consciousness, and Perceived ease of use significantly and positively impact online luxury purchase intention. Additionally, the findings indicated that brand awareness, perceived risk, and web atmospherics each intervened as moderators in the relationship between attitudes toward purchasing luxury products and online luxury purchase intentions. Product knowledge is not directly related to online purchase intention, but Attitude plays a mediating role in the relationship between product knowledge and online luxury buying purpose. In the context of luxury product intention, this study is one of the first to investigate the moderating effect that brand awareness, perceived risk, and web atmospherics play. It will help luxury brands develop the right tactics for selling luxury goods online in developing countries like Pakistan.

## 1. Introduction

Internet usage is continually enhancing due to technological advancements. The excessive involvement of consumers in online shopping has become an emerging trend now [1]. Consumers can shop for anything, from anywhere, just with a few clicks of fingers. The Internet has become the most efficient medium for selling all sorts of goods, varying from fast-moving consumer goods to luxury fashion goods [2]. However, for a long time, many organizations have not considered the Internet as a suitable medium for selling luxury goods [3]. The reason behind this is the concept known as the internet dilemma, defined as the difficulty faced by luxury firms to maintain their brand image, being exclusive in nature and simultaneously being available and accessible by anyone, anywhere [4]. Whether the Internet should only be used as an advertisement tool or a sales channel has been debated for a long [5]. Nevertheless, in today’s world, the Internet has been proven to be the quickest emerging medium for the sales of luxury items, as the luxury market has shown significant growth in the past twenty years [6]. Based on the current growth curve, analysts have forecasted that online luxury sales will generate a revenue of $85bn by the end of 2025, reaching from 10% sales in 2018 to 25% in 2025 [7].

The continuous increase in online luxury sales shows this segment has massive market potential. But the trend is lesser in emerging countries than in advanced countries because of the late adoption of internet-based services [8]. Consumers in Pakistan have not preferred online shopping of luxury products for long because of their concerns regarding product quality lack, rust, and satisfaction [9]. But also, according to some recent data, the youth of Pakistan is getting comfortable with online purchasing now; their increasing interest in shopping for luxury items online will give this market a new bloom [9]. Online purchasing has shown significant growth over the last few years in Pakistan. According to research conducted by Javed (2020), the E-commerce industry in Pakistan has at Rs. 51.8 billion in the fiscal year 2017, and the volume increased to Rs. 99.3 billion in the next fiscal year, 2018, exhibiting a growth of 92% [10]. The continuous evolvement in online payment infrastructures and online transactions has significantly changed this sector [11]. Although the online luxury sector is showing considerable growth in Pakistan, to the author’s best knowledge, no study has been done to study and comprehend the aspects that affect the online luxury purchase intention of customers in Pakistan. Many authors have conducted such research in Western countries, but these studies cannot provide a generalized view for the consumers of developing countries like Pakistan. This study tries to cover this study gap.

TAM (technology acceptance model) provides the foundation for this research. The primary emphasis of this research is to introduce a theoretical model based on TAM that determines the role of crucial variables that affect customers’ intentions to purchase luxury fashion products online [12]. According to the Reasoned Actions model, Technology Acceptance Model is the most extensively used model in describing consumers’ acceptance of information systems. This research adds new constructs to the existing technology acceptance model to understand consumers’ attitudes towards online shopping, especially in emerging economies like Pakistan [13].

The trend of online shopping is increasing in Pakistan at a fast pace. But the critical motivators after the virtual purchase of luxury fashion products in consumers in Pakistan are unknown [14]. This research aims to identify the role of crucial factors in shaping the Attitude of consumers of emerging economies toward purchasing luxury fashion products via the Internet. There are various Internet users in Pakistan, estimated to be 61.34 million in January 2021, and this number has increased by 11 million (+21%) between 2020 and 2021. Because of internet users’ enhancement, the trend of virtual shopping has also been increasing quickly. Revenue from E-shopping is expected to exhibit an annual growth of 5.51% by 2025 [15].

Moreover, the main aim of the research is to find out the crucial motivators after the virtual purchase of luxury-style products in consumers of Pakistan. Several studies have been done in the past years to understand the factors influencing the Attitude of web shoppers toward purchasing luxury fashion items [16]. But these studies do not provide a generalized view of the Attitude of web shoppers in developing economies like Pakistan. This study has applied the Technology acceptance model to online luxury consumption in Pakistan.

Luxury businesses are becoming more aware of the relevance of online platforms as an essential avenue for reaching out to consumers in developing nations and connecting with them [17]. It is becoming increasingly important to fully understand the elements that influence the consumers’ purchasing intentions in these marketplaces as the trend of buying online continues to gain steam [18]. This study comprehensively analyses the factors that shape consumers’ attitudes toward online luxury purchases in Pakistan. These factors include brand awareness, perceived risk, and web atmospherics. This study contributes to the existing literature by offering a nuanced understanding of the unique dynamics influencing online luxury purchase intention in developing economies. This is accomplished by examining various antecedent factors, such as product knowledge, price consciousness, perceived enjoyment, comfortability or perceived ease of usage, and usefulness.

Additionally, this study brings several contributions to the field. First, it investigates the mediating role of Attitude in the relationship between perceived usefulness, perceived ease of use, perceived enjoyment, and purchase consciousness. This helps shed light on the underlying psychological processes that shape consumers’ decision-making regarding online luxury shopping. Secondly, it explores the indirect impacts of product knowledge and price consciousness on purchase intention through Attitude, illuminating the subtle pathways through which these factors impact customers’ online luxury purchasing behaviour. In conclusion, this research investigates the moderating role of apparent threat, brand awareness, and web interference in attitudes toward luxury products and online luxury acquisition purposes. The findings of this study offer fresh perspectives on the contextual influences that shape consumers’ perceptions and attitudes in the online luxury marketplace. This research aims to inform policymakers, marketers, and luxury brands so that they can develop effective strategies and policies for navigating the rapidly growing online luxury market in developing economies like Pakistan. It does so by providing valuable insights into the aspects mentioned above of the market.

Even though online luxury shopping is becoming increasingly popular, there isn’t much study on what makes people in developing economies like Pakistan require to buy luxury products online. Luxury brands, policymakers, and marketers must understand these factors to navigate the online luxury market and create strategies that meet customer needs. So, this study tries to close the gap by examining how factors like product knowledge, price consciousness, perceived enjoyment, and perceived ease of use affect online luxury purchase intentions.

This study has important implications for both theory and practice. Theoretically, it adds to the existing research by analyzing the factors that affect how people in developing countries feel about buying luxury items online. The study reveals the psychological mechanisms that people’s decisions by examining how Attitude affects the relationship between perceived usefulness, perceived ease of use, perceived enjoyment, and buy intention. The study also looks at how perceived risk, brand awareness, and the atmosphere of the web affect consumers’ attitudes and perceptions in the online luxury market. This helps explain how context affects consumers’ attitudes and perceptions. The results of this study are helpful for luxury brands that do business in developing countries like Pakistan. When brands know what makes people want to buy something, they can change their marketing tactics and improve their online platforms to make it easier for people to buy luxury items. Policymakers can also learn from this study because it gives them helpful information for making the proper rules and policies to help the online luxury market grow in emerging countries, protect consumers, and create a thriving business environment.

Our paper follows the suggested guidelines, with an introduction summarising the research problem, a theoretical foundation section reviewing pertinent literature, a section where we develop and justify our hypotheses, and a section describing our research methodology. In this section, we analyze the results and discuss potential limitations and a section where we explore the theoretical and practical implications. The coherence of the information flow is ensured by this framework, which also facilitates understanding and assessment of our study.

## 2. Theoretical foundation

Luxury brands are essential in terms of their market value and very high growth rate. The luxury market’s growth is significantly higher than that of other categories of consumer goods [19]. In the words of Hagtvedt and Patrick (2009), "Luxury companies deliver high-priced products, and also deliver happiness as a crucial advantage and link with customers expressively" [20]. Moreover, according to Heine (2012), "Luxury brands are concerned with goods which focus on the ordinary and crucial than products of their group" [21]. Moreover, the functionalities of luxury companies are considered a status symbol, providing psychological value, and their user experience is highly related to a person’s self-esteem [22]. In 2014, attainment of greater than 1 trillion US dollars, the luxury marketplace comprised a vast range of categories, and its size is also appreciatable. The market is growing tremendously, with the total number of customers tripling over the past twenty years. This study emphasizes the virtual consumption of luxury fashion products because this is the fastest-growing online category [23].

As the monetary sales volume of the online luxury fashion market is increasing continuously, consumers’ wealth and increasing intentions for luxury consumption are the major boosters of the online luxury market [24]. Furthermore, with increased technology, various virtual fashion consumers are increasing rapidly because of the distinct features of E-shopping. It provides excellent product choices and convenience and ensures privacy and security [25]. According to another research, the Internet offers more product choices and price comparisons, making it easy to find anything online [26].

Online luxury fashion shoppers constitute skilled internet consumers and online purchasers [27]. A study classifies online consumers into three categories: online convenience, economic, and traditional shoppers. Consumers making online luxury purchases seek convenience [28]. Online shoppers are more educated and have more knowledge about brands and products. They have less brand loyalty and usually switch brands more frequently [29]. However, if the online store or website provides a pleasing and satisfying experience, it may convert them into repeat purchasers [30]. So, how online luxury fashion websites communicate and provide a purchase experience contributes to creating a new and disruptive experience for online purchasers, increases brand awareness, and ultimately provides a bridge to retaining customers [31].

According to the Reasoned Actions model, Technology Acceptance Model is the most extensively used model in describing consumers’ acceptance of information systems [32]. TAM proposes that an Individual’s reception of information schemes is mainly influenced by two factors, i.e., perceived ease of use (PEOU) and Perceived Usefulness (PU). The model was designed to illustrate technology usage [33]. Then it was prolonged and redesigned later, which aimed to utilize the expertise and was added as a mediator variable between independent variables, i.e., ’Perceived usefulness and perceived ease of use, and the dependent variable, ’use of technology. Various research has been conducted to find the attitudinal side of TAM [34]. It was also applied to study the impact of cultural differences on users’ attitudes toward technology. Later, TAM was prolonged to accept electronic commerce [35].

Much research has been carried out by adding new variables to this model. Some other researchers studied the cross-cultural comparison of Internet buying behaviour by adding new variables to TAM: internet usage, perceived risk, and innovativeness [36]. Similarly, some other researchers examined the predictive role of TAM by adding three additional constructs to this model, perceived enjoyment, user satisfaction, and perceived risk. Later on, the role of TAM was also examined in predicting consumer purchase intention in the luxury domain by adding four external variables: price consciousness, web atmospherics, perceived risk, and perceived enjoyment [37].

Consumer Online Purchase Intention is a widely studied subject of consumer cognitive behaviour [38]. According to the model of reasoned actions, consumer behaviour is directly predicted by a consumer’s intention related to the particular activity he is going to perform Purchase intention determines the desire of an individual to purchase a specific brand or product [39]. Purchase Intentions predict consumers’ likeliness to purchase in an online shopping environment. Another study depicts that to draw more consumers to the website, intention to purchase is more appropriate to consider than measuring consumer behaviour towards purchase. Online purchase intention is defined as the desire of a web shopper to buy goods from online retail stores with the help of the Internet [40]. The individual compares the prices of virtual products and then makes an actual purchase. Online purchase intention plays a vital role in determining accurate purchase decision-making, so deciding on the position of antecedent aspects that derive customers from making a purchase decision [41].

## 3. Hypotheses development

"An attitude represents the negative as well as the positive sensation of a person related to the performance of targeted behaviour. It is a person’s general feeling of favorableness and unfavorableness towards some stimulus object" [42]. Conferring to the reasoned action model, behaviour is forecasted by an intentional decision to execute that particular behaviour [43]. Furthermore, behavioural intention is predicted by a person’s Attitude towards the behaviour. Another research evaluated the mediating role of Attitude in predicting consumer intentions to shop online. It validated that Attitude toward the purpose of shopping online and online shopping is substantially connected—similarly, purchase intention formation across cultures by surveying US and Chinese consumers via structured questionnaires. The results were analyzed using the structured equation modelling technique, and their findings showed that emotions and Attitude play an essential role in creating consumer purchase intention for luxury brands [44].

Later, other researchers examined various factors determining Chinese consumers’ attitudes toward purchasing luxury fashion products. A total of 161 respondents were taken in three big cities in China, and by using regression analysis, his results indicated that Attitude toward purchasing luxury fashion items significantly affects Chinese consumers purchasing intention for luxury fashion items [45]. Another study reflected the customer behaviour toward luxury fashion products by collecting data from 257 real luxury consumers in India (Delhi) via a structured questionnaire, and data were examined through SEM and confirmatory factor analysis (CFA). The results suggested that Attitude is the second most crucial aspect that influences consumers’ purchase intention after subjective norms. Afterwards, based on a widespread literature review, a significant relationship between Attitude and purchase intention by commenting that purchase intention is influenced by consumers’ attitudes toward purchases. Similarly, another study explored the factors that influence the Attitude of Pakistani millennials toward green brands’ purchase intention. The authors concluded that Attitude toward certain products affects purchase intention under the theory of Reasoned Action [46].

Therefore, this study examines the role of Attitude as a mediator among online luxury purchase intention and preceding factors (namely, perceived risk, perceived enjoyment, product knowledge, perceived usefulness, perceived ease of use, price consciousness, brand awareness, and web atmospherics). Perceived usefulness is the extent to which individual trusts that utilizing a specific scheme would increase the job presentation of a person [47]. According to the current study, this definition means to what extent an individual finds virtual shopping more beneficial than a brick-and-mortar store.

Perceived usefulness is optimistically connected to virtual purchase intention. Similarly, perceived benefit is not crucially concerned with online purchase intention. People are more affected by the practicality of the actual product rather than the service of the way of shopping [48]. According to them, online shopping is feasible for low-cost and familiar products instead of high-involvement luxury products, as the latter usually involves money. Nevertheless, the availability of a wide variety of products and brands exclusively on the Internet also contributes to the perceived usefulness of online shopping. The ease provided to shoppers by the e-stores in making better purchase decisions also pertains to PU. Later, some other scholars researched to discover the influence of perceived usefulness on insolence to the online acquisition and concluded that PU influences attitudes toward an online purchase in a lively manner. Similarly, internet shopping gives shoppers more excellent product choices than physical stores, allowing them to compare different available brands and choose the best fit for them just with a few clicks of fingers [49, 50].

Similarly, a statistically positive association existed between PU and consumers’ behavioural intention to purchase online. In another study [51], PU has a direct optimistic association with purchase intention. PU is high in online purchases as it improves shoppers’ shopping ability and provides them with more excellent product choices. Therefore, the proposed hypothesis is as follows;

**H1:** *Perceived usefulness significantly influences consumer online purchase intention for luxury fashion products*.
**H2:** *Attitude towards purchasing luxury fashion products mediates the association between perceived usefulness and online luxury purchase intention*.

Perceived Ease of Use (PEOU) refers to how individual trusts utilizing a specific scheme would remain independent of hard work [52]. According to the current study, this description denotes a person’s perception that purchasing luxury fashion products online requires minimal effort. PEOU is very important in the case of internet shopping, as it is concerned with the user-friendliness of a particular website of the brand. If the website is poorly designed and takes a long time to reply and load the required information, it will cause the online shopper to lose interest, and he will probably go for conventional shopping channels. So there is a happy relationship between the comfortability of the usage of technology and purchasing purpose of the consumer [53].

Moreover, another researcher [54] studied the mediating role of Perceived ease of use in the electronic purchase intention of Taiwanese customers and stated that PEOU significantly influences online purchase intention. They argued that PEOU has an additional optimistic influence on purchase intention when the website is easily accessible, and processes are straightforward, i.e., consumers face fewer complications while placing orders. It is the chief factor contributing to the virtual store’s success [55].

While evaluating the influence of perceived ease of use on consumers’ attitudes towards online luxury purchase intention in Indian consumers, Jain (2020) stated that there is a direct association between purchase intention and PEOU. He commented that physical luxury retail stores are only built-in in larger cities in India [18]. So, online luxury fashion stores enable consumers to shop for luxury fashion goods anytime, anywhere, even from the country’s smallest towns. Hence, we proposed the following hypothesis;

**H3:** *Perceived ease of use influences consumer online purchase intention to buy luxury fashion products*.
**H4:** *Attitude towards purchasing luxury fashion products mediates the association between perceived ease of use and online luxury purchase intention*.

The other factor that influences consumer attitude towards online luxury fashion consumption is product knowledge (PK). "Product knowledge has become an important part of consumer behaviour. It is all about awareness of customers regarding particular data that is all about a given product." [56]. The two main bases of product information are the customer’s prior involvement with the good or the advertisement designed to encourage the consumer to make a purchase decision. So, a consumer’s awareness regarding the product or his confidence in the product gives rise to product knowledge. In addition, consumers’ knowledge about the attributes and features of the product, its kinds and categories, cost of the product, trust in the product, and related benefits and satisfaction provided by the product all constitute product information [57].

Moreover, some other researchers explored the impact of product knowledge on customer purchase intention in catering amenities and insurance purchases in Taiwan. They concluded that product knowledge has a crucially optimistic effect on customer purchase opportunities [58]. In another research, "product knowledge" was taken as an independent variable and researched to evaluate its impact on purchase intention. The conclusion drawn from statistical output stated that consumer product knowledge influences purchase intention such that the higher the product knowledge, the higher the choice to buy a particular product. The research implied that businesses should provide complete product descriptions and information to influence consumer purchase intention positively [59].

[60] studied the influence of product knowledge on purchasing organic cotton through a survey that 500 Generation Y respondents led. They applied structured equation modelling to analyze the results, and independent t-tests were utilized to determine the impact of one construct over the other. Based on the findings, the study confirmed the influence of product knowledge on purchase intention. According to them, product knowledge strongly affects consumers’ attitudes toward purchasing a particular product. Zhuang and colleagues (2021) provided insight into how green product information affects and encourages green purchase intention. Based on an analysis of 236 samples, the study concluded that green product knowledge directly predicted purchase intention towards green products and assumed that customers with higher product knowledge are more likely to purchase those products [61]. The following hypothesis is proposed after a detailed review of the literature;

**H5:** *Product knowledge significantly influences consumer online purchase intention towards luxury fashion products*.
**H6:** *Attitude towards purchasing luxury fashion products mediates the association between online luxury purchase intention and product knowledge*.

Perceived Enjoyment (PE) denotes utilizing a particular scheme that can be attained to remain relished in its way apart from the outcomes of presentation that can result from using the method. Clients shop virtually for "arousal, fun, enjoyment, and sensory stimulation" [62]. According to some typologies, consumers do shopping to achieve specified goals with minimum effort. It is termed a utilitarian view. On the other hand, one customer said, "I adore looking around and imagining what I might buy if I ever had enough cash. Shopping is a thrilling experience.". It deliberates that shopping is a possible source of adventure and fun rather than achieving a specified goal [63].

While studying the Explored hedonic motivations for online shopping behaviour, enjoyment strongly predicts consumers’ attitudes toward online shopping [64]. If online shopping is enjoyable for consumers, they will develop a more positive attitude toward web-based shopping, and consequently, they will accept the Internet as a shopping standard. Enjoyment felt by the consumer in shopping online directly influences consumer purchase intention. Perceived enjoyment is crucial in determining online purchase intention [65]. They further concluded that making websites enjoyable would benefit virtual businesses. However, some other researchers examined the influence of shopping orientations on customer online purchase intentions in Malaysia, and the findings were not aligned with the past research. They rejected the hypothesis that "shopping enjoyment is optimistically concerned with online purchase intention." Moreover, they concluded that perceived enjoyment is not significantly connected to consumer online purchase intention in a virtual setting [66].

In contrast, another research concluded that many customers shop for luxury products to contain their emotional needs, i.e., they seek enjoyment. Researchers conducted controlled sampling, and questionnaires were distributed to actual users of luxury fashion brands through personal interviews. The hypothesis was examined through structural equation modelling using AMOS. The statistical analysis showed that enjoyment is a strong forecaster of purchase intention for luxury products. They added that consumers enjoy the notion of keeping expensive luxury items. Jain (2020) found that perceived enjoyment directly affects the online purchase intention of customers. The author also found an indirect association between PE and PI via Attitude [6, 18]. The findings disclosed that consumers go shopping for luxury fashion products to complete their fun-pursuing requirements somewhat more than practical requirements. So, it is hypothesized that;

**H7:** *Perceived enjoyment significantly influences consumer online purchase intention*.
**H8:** *Attitude towards purchasing luxury fashion products mediates the association between perceived enjoyment and online luxury purchase intention*.

The other factor influencing consumer attitude significantly is Price consciousness (PC). Price is one of the most common aspects that shape customer purchase intention, especially online luxury purchasing. Consumers are checking and comparing the costs of different luxury brands to get better deals as they aim to purchase expensive and flagship luxury items at the best prices. As discounts are attractive, consumers shop online rather than in brick-and-mortar stores. While contrasting the in-store and online shopping behavior of customers to luxury products, one of the consumers said, "If the quality is just like this and I can be confirmed about the appropriate match, and I can relish the minimum cost and ease of internet seller. Later, some other researchers looked at the factors that influence the online shopping decisions of consumers. They analyzed the advantageous factors that drive consumers to shop online, like fast delivery, low and comparable prices, and more extensive choices [67].

The research was conducted by analyzing data collected via an online questionnaire from 183 online shoppers. Empirical findings of the study showed that online shoppers are mainly affected by the convenience of the shop, better prices, and the possibility of comparing prices. Jain (2020) examined the role of various factors that shaped consumers’ behaviour in shopping for luxury fashion items online and showed that PC is positively concerned with online buying intention. The author indicated that better prices positively affect consumer attitudes toward purchasing luxury goods online [18]. Hence, So, it is hypothesized that;

**H9:** *Price consciousness significantly influences consumer online purchase intention for luxury fashion products*.
**H10**: *Attitude towards purchasing luxury fashion products mediates the association between price consciousness and online luxury purchase intention*.

This study also has three moderating variables to get detailed insight into factors affecting online purchase intention. First, it will examine the role of Brand Awareness in shaping consumers’ attitudes. A Brand is defined as a sign, name, term, design or symbol, or a consolidation of these elements to recognize the products and services of one selling entity or a group of trading commodities and distinguish them from those of competitors [68]. And brand awareness is connected to the power of the node of the brand in reminiscence of customers, and it can also be deliberated by the capability of customers to identify the company in numerous circumstances or contexts" Brand awareness is comprised of two main elements, i.e., Recognition of a brand, recalling a brand [69].

Moreover, brand recognition refers to customers’ ability to ensure contact with the company once it delivers a company as a signal. Brand recall refers to the probability of continuous remembrance of a brand name through a customer once encouraged with service or product or any connection with it. It is also an effect of customers’ capability to recall the brand name [70]. The brand choice of a consumer is affected by brand awareness. Purchase intention is higher for the brands the consumer is aware of, and when a consumer has information about the brand, he perceives it to be a good brand.

To examine the influence of Brand awareness on consumers’ purchase intention, some researchers researched Taiwan by collecting information from cellular phone users. The data was analyzed through regression testing. It was determined that there is a crucial positive association between consumer purchase intention and brand awareness, i.e., consumers tend to buy cell phones of only those brands with which they are familiar [71].

Another researcher studied the mediating role of brand awareness in acquisition intention for products of high involvement. The author applied structured equation modelling to analyze the results and commented that brand awareness indirectly and positively affects consumer intention to purchase. Another researcher studied how brand awareness and electric word of mouth affect buying intention in the brand image presented as a mediator. The research was conducted by collecting data through 300 questionnaires in Rawalpindi and Islamabad. After co-relation and regression testing, results showed that consumer purchase intention is positively affected by brand awareness [71].

Using a qualitative research approach, the authors elaborated the influence of brand awareness on customer purchase intention [72]. After reviewing various research papers and articles from 2000 to 2016 on the influence of brand awareness on buying intention, the authors concluded that customers are likely to purchase those brands they know. Luxury products involve considerable money, so consumers hesitate to buy them from a new or unfamiliar brand. In addition, consumers tend to do market research to get well aware of the brand they will shop from. Therefore, the following hypothesis is proposed:

**H11:** *Brand awareness moderates the influence of Attitude on customers’ intention to purchase luxury fashion products*.

The study also adds PR as a moderating variable on the association between Attitude and consumer purchase intention. Perceived risk is a "customer’s perceptions of the kind and extent of uncertainty once considering buying decision". Some other researchers determined the features that derive customers from shopping online. According to the authors, online shopping is risky, as consumers cannot physically touch and feel the product. People are reluctant to shop online because they do not find sharing their financial and personal information comfortable with different companies. Therefore, risk plays a regulating role in online purchase intention and customers’ attitudes. Consumer trust violations, misuse of personal information, and privacy breaches negatively affect attitudes toward online purchase intention [73].

Some other researchers researched to explore the influence of online presentation, mood, and perceived risk on online purchase intention. The scholars concluded that perceived risk adversely impacts online purchase intention, i.e., the more advanced the perceived risk, the inferior the intention to shop online will be. The shopper must be confident in the brand or product to purchase. Providing a 3-dimensional view of the product and a comprehensive product description may build shopper’s confidence, positively affecting purchase intention [74].

Another researcher compared the physical and online shopping behaviour toward luxury products. The qualitative study concluded that consumers are unwilling to shop virtually due to the inherent risk of internet shopping. Perceived risk is higher for luxury goods as they involve a more significant sum of money. So online retailers should provide quality customer service and maintain product quality so that consumers provide positive ratings and reviews, ultimately leading to higher buying purposes for other incoming shoppers [75]. There is also a concern that shoppers fear purchasing luxury products online. They wish to physically touch and feel the product before buying because luxury products represent one’s self-identity, and their quality cannot be compromised [18]. Jain (2020) considered the moderating part of perceived risk in online luxury purchase intention. The author stated that risk perceived by customers moderates the influence of Attitude on buying intention in such a way that the lower the perceived risk, the higher the consumers’ Attitude toward purchasing luxury fashion products. The following hypothesis is made after reviewing the literature;

**H12:** *Perceived risk controls the influence of Attitude on consumers’ intention to buy luxury fashion products online*.

Web atmospherics is "the purposeful design of the retail environment to have a better impact on the customer" Website quality and atmospheric cues play a very crucial role in online retailing. Another researcher suggested that the online store atmosphere, like merchandise colors, presentation styles, and easy navigation, is strongly linked to the development of consumer purchase intention. The atmosphere does not completely regulate purchase intention, but consumers are four times more likely to purchase if web atmospherics are promising [76]. Online store atmospherics are essential to attract and retain online customers. Another research proposed web atmospherics as a marketing tool that creates a positive purchasing environment [77].

**Fig 1:**
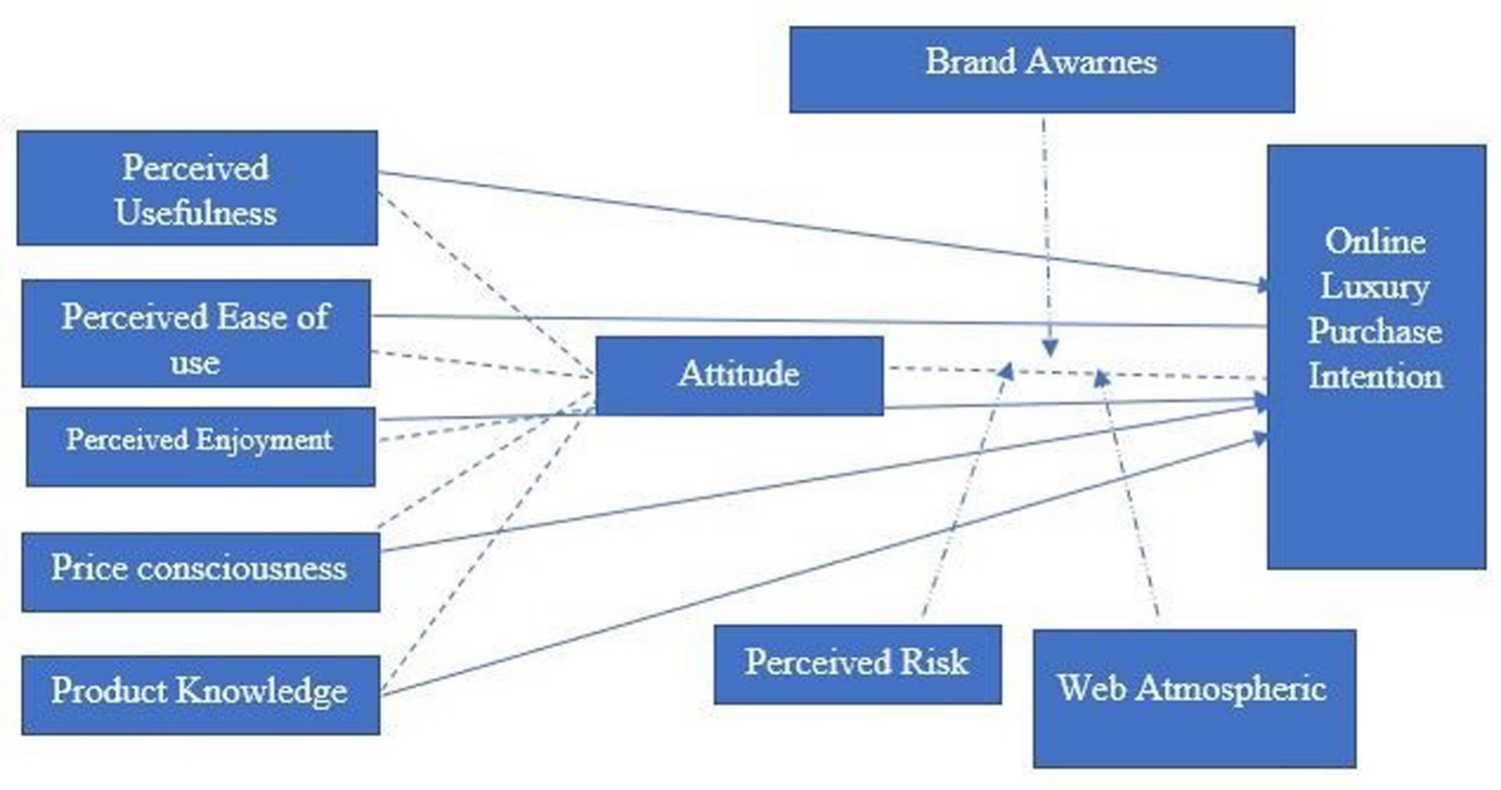
Conceptual framework of the study.

The online purchase behavior was studied using two different website designs for a virtual retail store, and Chinese and American university students’ shopping attitudes were examined. The results showed that a well-designed customer interface and quality website positively affect consumer attitude towards purchase [78]. Customers are more likely to be attracted to a visually appealing website and develop positive purchase intention. Ha and Lennon (2011) created two fake websites to examine consumer responses to the online atmosphere. One website contained atmospheric cues like good graphics, coloured icons, background patterns, and a catchy brand logo, though the other had only a simple logo and hyperlinked texts. Both websites included the same product and product description, and a random sample of 148 students was allowed to purchase through any of the websites. The findings demonstrated that affective atmospheric cues reduce the perceived risk, attract consumers’ attention and consequently lead to the decision to make an online purchase [79]. Roberts and Grassi (2021) suggested that atmospheric cues like zoomed images, 360-degree view of the product, and videos of models using or wearing luxury products provide in-depth information to online shoppers, which assists them in making an online purchase decision. Easy-to-navigate websites will also strengthen the consumer attitude toward purchase intention [80]. Jain (2020) examined the moderating role of web atmospherics in online luxury purchase intention and found that Purchase intention is meaningfully affected by web atmospherics [18]. Fig 1, represent the conceptual framework of the study. The higher the website is visually attractive, the higher the consumer purchase intention towards luxury items. The following hypothesis was derived;

**H13:** *Web atmospheric quality moderates the effect of Attitude on the intention of customers to buy luxury fashion products online*.

Online luxury purchase intention is a multifaceted phenomenon influenced by various factors, with perceived usefulness being a central determinant. Building on the Technology Acceptance Model (TAM), scholars argue that perceived usefulness, defined as the degree to which an individual believes that using a particular system would enhance their performance, significantly impacts online purchase intention [81]. In the context of luxury goods, consumers are likely to develop an intention to purchase online when they perceive that the digital platform enhances their overall luxury shopping experience. Recent studies, such as [82], have affirmed the positive relationship between perceived usefulness and online purchase intention, emphasizing its relevance in understanding consumer behavior in the luxury segment [83].

Perceived ease of use is another critical factor influencing online luxury purchase intention. Stemming from TAM, this concept refers to the degree to which a person believes that using a particular system would be free from effort [84]. In the context of luxury goods, consumers are more likely to express an intention to purchase online if they perceive the online platform to be user-friendly and easy to navigate. Another Research [85] found a positive association between perceived ease of use and online purchase intention, highlighting the importance of a seamless and effortless online shopping experience in the luxury market.

Perceived enjoyment is an emotional aspect that significantly shapes online luxury purchase intention. Stemming from the theory of hedonic motivation, perceived enjoyment refers to the pleasure and satisfaction derived from using a particular system [86]. In the realm of luxury goods, consumers are likely to exhibit a higher intention to purchase online when they derive enjoyment from the digital shopping experience. Studies, such as that by [87] highlight the positive impact of perceived enjoyment on online purchase intention in the luxury context, emphasizing the role of emotional satisfaction in driving consumer behavior.

Price consciousness is a pragmatic factor influencing online luxury purchase intention. Consumers with a high level of price consciousness are driven by a keen awareness and sensitivity to the costs associated with luxury products [88]. In the online environment, consumers may be more inclined to purchase luxury items if they perceive the online platform as offering favorable pricing, discounts, or added value. This aligns with findings from research by [89] which underscores the significance of price consciousness in shaping consumer behavior in the luxury e-commerce space. Product knowledge plays a unique role in shaping online luxury purchase intention. While it might not directly impact intention, it can act as a mediating factor. Consumers with high product knowledge may not be directly inclined toward online luxury purchases; however, their attitude toward such purchases is likely to be influenced by their knowledge [90]. This is supported by research like that of [91],which highlights the mediating role of attitude in the relationship between product knowledge and online luxury purchase intention.

Perceived risk is a critical moderating factor influencing the relationship between attitudes and online luxury purchase intention. In the context of luxury goods, perceived risk, which encompasses concerns about the potential drawbacks or uncertainties associated with online purchases, can either amplify or mitigate the impact of consumer attitudes on their intention to purchase online. Research by Kim and Forsythe (2009) indicates that consumers with higher perceived risk may exhibit a weakened relationship between their attitudes toward online luxury purchases and their actual purchase intentions [92].

Web atmospherics, including website aesthetics, design, and interactivity, play a pivotal role in shaping online luxury purchase intention. A visually appealing and immersive online environment enhances the overall shopping experience, positively influencing consumers’ attitudes and intentions [93]. Another Research supports this, suggesting that a well-crafted web atmosphere strengthens the link between consumer attitudes and their intention to purchase luxury items online [94]. Brand awareness is a crucial moderating factor influencing the relationship between attitudes and online luxury purchase intention. In the luxury context, consumers with higher brand awareness may exhibit a stronger link between their attitudes toward online purchases and their actual purchase intentions [95]. Similarly, Another study highlighted the role of brand awareness in reinforcing positive consumer attitudes and intentions in the online luxury market [96].

## 4. Methodology

This study is mainly conducted in Pakistan to see whether brand awareness, perceived risk, and website design significantly influence the insolence of Pakistani consumers towards the online purchase of luxury fashion products. Both males and females use luxury fashion products, so research is not stereotyped to only one gender The study examines the factors influencing the online buying intention of Pakistani customers. The population of Lahore has been targeted for this purpose. Lahore is one of the wealthiest cities in Pakistan, the capital of the biggest province of the country, with estimated GDP of $84 billion in 2019. That’s the reason people living in Lahore are assumed to shop for luxury fashion products online.

It is essential to know which aspects encourage people to buy luxury fashion products virtually and which factors negatively or moderately affect them. The respondents were selected after qualifying the questions like "Do you use luxury fashion products?". The questionnaire was shared with only those who said "Yes" upon asking the question. The participant took formal consent. A written consent was taken through a consent form (Attached in S1 File). The study was conducted according to the guidelines of the Declaration of Helsinki. Ethics approval was sought by the University of the Punjab, Pakistan (ethical approval no: IBIT/RC/202134)

A single population element representing all the potential target respondents is known as a sampling unit. A purposive sampling technique has been used in this research, called judgmental sampling, in which the selection of respondents is made based on the sound judgment of the researcher. Unfortunately, the actual population of luxury fashion consumers in Lahore was unknown, so the sample size could not be measured. According to [97], a sample size greater than 200 is less likely to show data normality issues. So, a link for this survey was shared with the people living in Lahore only, and a total of 350 responses were gathered, from which 83 answers were eliminated and 267 responses were considered for further analysis. The language of the questionnaire was English, and information regarding demographics, web atmospherics, perceived risk, perceived shopping enjoyment, perceived usefulness, product knowledge, perceived ease of use, price consciousness, and brand awareness was collected.

Existing scales have been adopted to develop the questionnaire for this research. One of the most popular scales developed by Ajzen and Fishbein (1980) [98] and Jain et al. (2017) was used to measure Purchase Intention [99]. Childers and his colleagues (2001) adopted one of the most popular scales to evaluate Attitude [100]. Scales developed by Shukla (2014) [101] and Zheng and colleagues (2012) were used to measure perceived risk [102]. The scale for brand awareness was adopted from Yoo & Donthu’s (2001) study [103].

### 4.1 Data analysis and results

The descriptive analysis examines essential fundamental attributes of information gathered from respondents. Characteristics of demographic profiles of participants are one of the vital components of data analysis; it allows managers and policymakers to generalize their results and formulate policies accordingly. The study’s first segment holds respondents’ demographic profiles like monthly household revenue, education, marital status, gender, age, and proficiency on the Internet. Characteristics of demographic profiles are analyzed and presented in Table 1 (Detailed data available in S1 Table). Results show more female participants than male participants, as out of 267 responses, 154 were females. Most of the participants were young and fit into a group of 20 to 25 years old. Regarding education, most of the respondents have completed their graduation and Master, i.e., out of 267 respondents, 138 were graduates, and 106 respondents have done their Master’s or MPhil degrees. Only two illiterate people participated in this research.

**Table 1:** Demographic distribution of the respondents.

VARIABLES	CATEGORIES	FREQUENCY	PERCENTAGE
**Gender**	Male	113	42.3
	Female	154	57.7
**Age**	Less than 20 years	14	5.2
	20 to 25 years	165	61.8
	26 to 30 years	59	22.1
	31 to 35 years	11	4.1
	36 to 40 years	4	1.5
	Above than 40 years	14	5.2
**Education**	literate	2	.7
	Matriculation	4	1.5
	intermediate	15	5.6
	Graduate	138	51.7
	Masters/MPhil.	106	39.7
	PhD	2	.7
**Marital Status**	Single	206	77.2
	Married	61	22.8
	Divorced	0	-
	Widow	0	-
**Occupation**	Student	122	45.7
	Employed	82	30.7
	Unemployed	26	9.7
	Businessman	13	4.9
	Housewife	24	9.0
**Monthly Household Income (PKR**	Below 50,000	92	34.5
	50,000 to 79,999	57	21.3
	80,000 to 109,999	40	15.0
	110,000 to 139,999	31	11.6
	140,000 and above	47	17.6
**Proficiency on the Internet**	Not skilful	13	4.9
	Somewhat skilful	85	31.8
	Skilful	125	46.8
	Very skilful	44	16.5

Moreover, 77.2% of the respondents were single, the rest were married, and no were divorced or widowed. Regarding profession, 45.7% of respondents were students, while 9.7% were unemployed. The rest of them were Employed, business people or homemakers. Most respondents had an income below Rs. 50,000 (34.5%), maybe because most participants were students. 46.8% of respondents were skilful in Internet use, while only 4.9% were non-skilled internet users.

### 4.2 Measurement model assessment

In the first phase of the reflective measurement model assessment, internal consistency reliability is measured with the help of Cronbach’s alpha and composite reliability. Composite dependability is favored above Cronbach’s alpha in PLS-SEM. It ranges from 0 to 1, with a higher number indicating more excellent composite reliability. Values of 0.6 to 0.7 are generally perceived as acceptable and reliable [97]. Internal consistency is less dependable, given that the value is less than 0.6. Table 2, represent the Composite Reliability

**Table 2:** Internal consistency reliability.

Variables	Cronbach’s alpha	Composite Reliability
Online Purchase Intention	0.910	0.930
Attitude	0.908	0.905
Perceived Usefulness	0.878	0.898
Perceived Ease of Use	0.897	0.869
Perceived Enjoyment	0.930	0.940
Price Consciousness	0.829	0.819
Product Knowledge	0.867	0.847
Brand Awareness	0.927	0.917
Perceived Risk	0.712	0.712
Brand Awareness	0.927	0.917
Web Atmospherics	0.871	0.881

The model’s validity was assessed using the Heterotraite-Monotrait Ratio. According to the data, all the HTMT values are lower than the required threshold of (0.129–0.627 0.85), indicating the model’s discriminant validity has been validated [104]. Table 3, represent the HTMT values of the model.

**Table 3:** Heterotrait-Monotrait Ratio (HTMT).

	AT	BA	PC	PE	PEOU	PE	PK	PR	PU	WA
**AT**	0.909									
**BA**	0.372	0.857								
**PC**	0.483	0.545	0.776							
**PE**	0.468	0.489	0.615	0.917						
**PEOU**	0.502	0.558	0.631	0.657	0.830					
**PE**	0.622	0.420	0.480	0.578	0.456	0.903				
**PK**	0.542	0.675	0.595	0.615	0.533	0.577	0.806			
**PR**	0.453	0.453	0.440	0.534	0.464	0.456	0.546	0.708		
**PU**	0.611	0.505	0.611	0.656	0.670	0.608	0.672	0.580	0.863	
**WA**	0.432	0.657	0.612	0.562	0.594	0.466	0.525	0.407	0.575	0.844

The convergent validity of reflective variables was assessed using the AVE method. Convergent validity refers to the degree to which various measures of the same construct are positively correlated. An AVA value of 0.5 or higher, according to Hair et al. (2012), describes more than 50% of the variance of its indicators [97]. Table 4 indicates that all the AVE values are above the threshold value of 0.50. Perceived enjoyment has the highest AVE value of 0.840, while Perceived Risk has the lowest AVE value of 0.502 respectively; hence, both values are above than critical value.

**Table 4:** Average variance extracted.

Variables	Average Variance Extracted
Online Purchase Intention(OLPI)	0.815
Attitude(AT)	0.826
Perceived Usefulness(PU)	0.745
Perceived Ease of Use(PEOU)	0.689
Perceived Enjoyment(PE)	0.840
Price Consciousness(PC)	0.602
Product Knowledge(PK)	0.649
Brand Awareness(BA)	0.735
Perceived Risk(PR)	0.502
Web Atmospherics(WA)	0.713

### 4.3. Structural model assessment

The path coefficient represents one variable’s direct and moderating effect on another. It also reveals the strength of their relationship. A value around 1 indicates a greater correlation, whereas a value near zero indicates a weaker association. Near-zero values are not statistically significant [97]. Bootstrapping was used to generate significance values to ascertain if the association was substantial. At a 5% significance level, the crucial value for two-tailed tests is p-value = 0.05. It can be seen from Table 5 that Attitude, Perceived Ease of use, perceived enjoyment, and Perceived enjoyment exhibit a significant positive association with Purchase intention as their significance value is greater than 0.05. But price consciousness, product knowledge, and perceived usefulness exhibit no association with Purchase Intention. Perceived usefulness, perceived ease of use, perceived enjoyment, and price consciousness exhibit significant positive associations with Attitude. Moreover, it can be seen that web atmospherics and brand awareness exhibit significant positive moderation over purchase intention. And perceived risk exhibits Significant negative moderation over purchase intention.

**Table 5:** Significance of path coefficient.

	Original Sample (O)	Sample Mean (M)	Standard Deviation (STDEV)	T Statistics (|O/STDEV|)	P Values
**AT -> PI**	0.624	0.621	0.118	5.297	0.000
**BA -> PI**	0.520	0.507	0.077	6.730	0.000
**Moderating Effect BA -> PI**	0.280	0.275	0.047	5.971	0.000
**Moderating Effect WA -> PI**	0.141	0.139	0.038	3.759	0.000
**Moderating Effect PR -> PI**	-0.234	-0.235	0.042	5.518	0.000
**PC -> AT**	0.178	0.179	0.083	2.150	0.032
**PC -> PI**	-0.013	-0.008	0.056	0.232	0.817
**PE -> AT**	0.226	0.227	0.069	3.261	0.001
**PE -> PI**	0.177	0.168	0.057	3.130	0.002
**PEOU -> AT**	1.296	1.297	0.077	2.759	0.000
**PEOU -> PI**	0.304	-0.311	0.073	4.185	0.000
**PK -> AT**	0.231	0.238	0.084	2.757	0.006
**PK -> PI**	0.112	0.116	0.067	1.674	0.095
**PU -> AT**	0.841	0.847	0.113	7.422	0.000
**PU -> PI**	0.136	0.150	0.120	1.129	0.259
**WA -> PI**	0.095	0.090	0.035	2.714	0.007

The independent variables create variability in the dependent variables, as shown by R2. It has a value of 0 to 1. A value near 1 suggests greater accuracy than a number around 0. According to [10];R^2^ = 0.67 is strong,R^2^ = 0.33 is moderate and R^2^ = 0.19 is weak. Table 6, represent the R squares values of the variables.

**Table 6:** Coefficient of determination (R square value).

	R Square	R Square Adjusted
**AT**	0.804	0.702
**PI**	0.823	0.753

**Fig 2:**
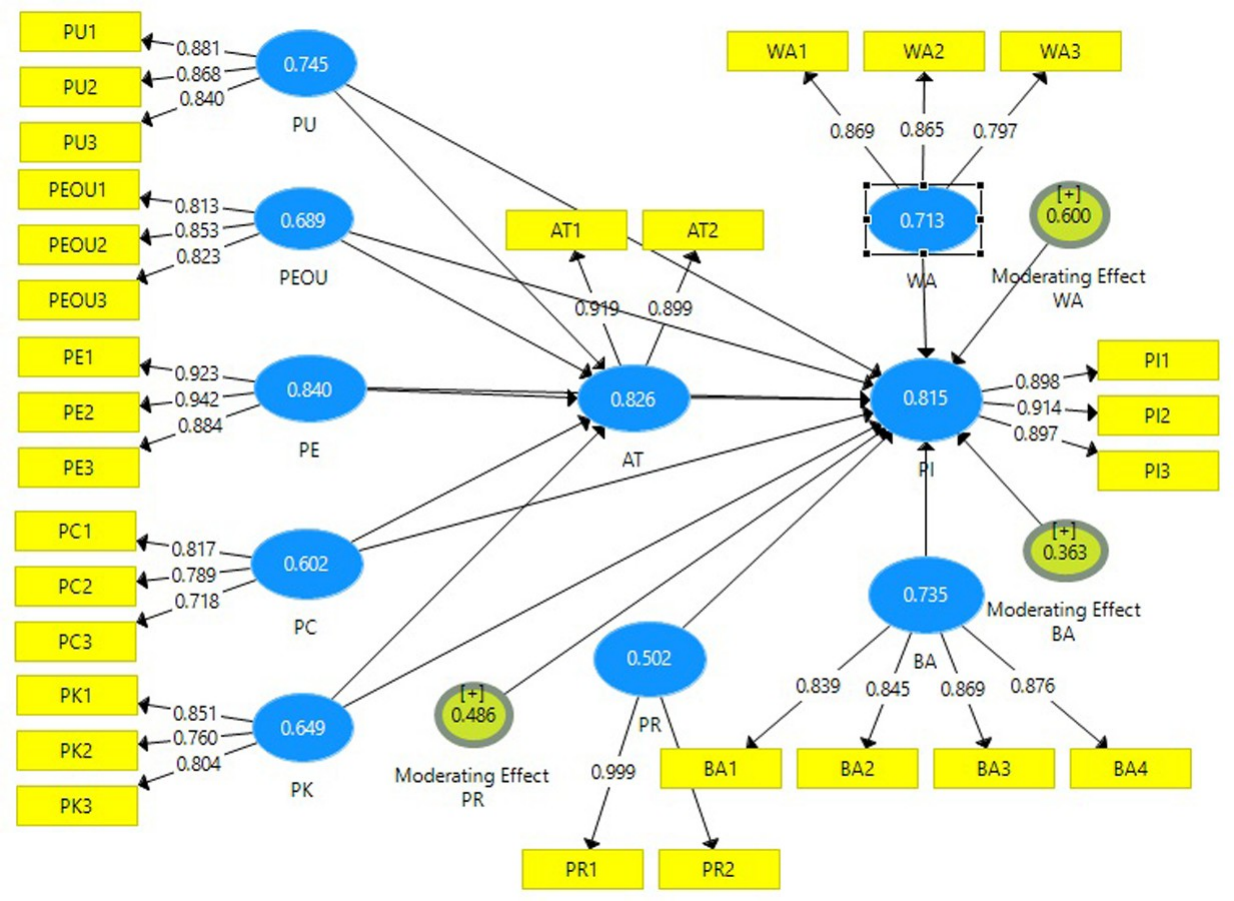
SEM-Model of the study.

Whereas adjusted R^2^ is a modified form of R^2.^ The adjusted R^2^ is mostly less than R^2^ values (Hair et al. 2014). Values of Adjusted R^2^ and R^2^ are displayed in the Table 6.^.^ The R2 of the dependent variable Attitude is 0.804, and the conditional inconsistent Purchase Intention is 0.823, which is considered a strong effect. Moreover, the adjusted R^2^ values are less than the R^2^. Similarly, Fig 2 depicts the SEM model of the study.

## 5. Discussion

This research aims to determine how various factors like perceived enjoyment, price consciousness, perceived usefulness, perceived ease of use, and product knowledge accentuate consumers’ Attitudes toward online luxury purchase intention. The research also aimed to assess the moderating role of risk perceived in online shopping, brand awareness, and web atmospherics in attitudes towards luxury products and intention to purchase luxury products online. To accurately analyze the different components of the research, a sample of 267 premium fashion consumers are gathered through an online questionnaire utilizing the purposive sampling technique.

SPSS is used to explore the differences ascending due to demographics, and PLS-SEM is utilized further to evaluate the existing link between dependent and independent variables. Demographic analysis shows that female respondents were comparatively higher than male respondents constituting 57.7% of the entire sample. The myriads of respondents are from 20–25 years, forming 61.8% of the whole piece. Most respondents were graduates and Masters/MPhil, making up 51.7% and 39.7% of the sample. The demographic profile also included marital status, which showed that 77.2% of respondents were single.

Regarding occupation, a significant proportion of the respondents were either students or employed, forming up 45.7% and 30.7% of the entire population sample. A considerable portion of respondents (34.5%) earn below Rs. 50,000 monthly. It also included proficiency on the Internet. A significant percentage of 46.8% of respondents was skilled in using the Internet.

Results obtained from PLS-SEM exhibited a strong coefficient of determination, R2 = 0.804 for Attitude and R2 = 0.823 for online purchase intention, which indicates the variation in the dependent variables, Attitude, and online purchase intention, is because of the independent variables. The value R2 = 0.804 and R2 = 0.82 R2 = 0.823 indicates 80% and 82% of Attitude toward online purchase intention, following independent variables, respectively. Path coefficients were also analyzed after running 5000 bootstrap samples in PLS-SEM, and the results exhibited that perceived usefulness was not directly linked with online purchase intention. These results are the same as obtained from the previous work by different researchers [73], which found that perceived usefulness is unrelated to online purchase intention. Individuals are more affected by the usefulness of the actual product compared to the usefulness of the way of shopping [31, 69].

Similarly, according to the results, product knowledge did not hold any direct link with online purchase intention. The findings do not support the prior work done by the business scholars [9, 19, 73], as according to these researches, product knowledge influences purchase intention, and it holds a strong and directly positive link with purchase intention. At the same time, perceived enjoyment, ease of use, and price consciousness strongly correlated with online purchase intention. The results obtained from the collected data and multiples statistical analysis match the prior studies done by some well-known previous research [45, 56, 72].

The following mediation effect was examined, and path coefficients were analyzed to determine the indirect effect of all the variables, such as perceived ease of use, perceived usefulness, price consciousness, product knowledge, and perceived enjoyment, on online purchase intention through Attitude. All the antecedent factors were found to have a significant indirect association with online purchase intention. These findings follow the previous research conducted by [4, 5, 31].

Brand awareness is a vital part of how people decide what to buy. Another recent study shows how vital brand awareness influences consumers’ buying decisions [105, 106]. The study found that when people know more about a brand, they have more good feelings about it and are likelier to buy it. Similarly, another research found that brand awareness positively moderates the relationship between attitude and purchase intentions. Consumers with higher brand awareness are more likely to turn their positive attitudes into actual purchases. The moderating role of brand awareness was examined, and results indicated that brand awareness is positively linked with Attitude and purchase intention such that the more brand awareness, the higher the Attitude toward online purchase intention. These findings align with the arguments present in the literature [17]

Another critical factor that affects what people buy online is how risky they think it is. Recent studies have shown that how customers feel about risk affects their decision to buy something online. For example, Ellis-Chadwick’s and Doherty (2012) study found that higher levels of perceived risk make people less likely to buy online [107]. Also, a study found that people think there are more risks when buying luxury goods online, which makes them less likely to buy. Based on the data analysis and previous studies, perceived risk significantly moderates the relationship between Attitude and intentions to buy luxury items online [42].

The quality of website design, layout, and interactivity, among other things, has been a critical factor in how consumers feel and what they plan to buy online. A recent study has taught us much about how web atmospheric quality acts as a moderator. For example, Li and Zhang’s study from 2021 showed that a good web experience makes people more likely to like the company and make a buy. Similarly, Sarah et al. (2021) found that the quality of web atmospheric elements affects consumers’ intentions to buy online, especially regarding high-end goods. Lastly, the moderating effect of web atmospherics was analyzed, and results showed that web atmospherics positively moderates the link between online purchase intention and the Attitude of the buyers such that the better the web atmospherics, the higher will be the Attitude toward online purchase intention [77]. These findings align with the arguments in the multiple research projects studied by eminent scientists and business scholars [4, 108].

This research stands out for its distinctive contribution to the field by intricately exploring the facets of online luxury purchase intention within the intricate market landscape of developing nations, notably Pakistan. Setting itself apart from previous studies, the research meticulously scrutinizes a spectrum of factors, including product knowledge, price consciousness, perceived enjoyment, perceived ease of usage, and usefulness, thereby offering a more nuanced comprehension of the dynamics at play in the Pakistani luxury fashion market. This not only enriches the broader discourse on online consumer behavior but also fills a critical void in the understanding of luxury consumption within emerging economies. The innovative aspect of the study is further underscored by the inclusion of moderating variables such as perceived risk, brand awareness, and web atmospherics, providing a sophisticated lens through which to examine the intricate interplay between consumer attitudes and online luxury purchase intentions. Consequently, this research doesn’t merely contribute to academic literature but also furnishes industry stakeholders with valuable and distinctive insights, guiding the strategic development of online luxury retailing practices uniquely tailored to the specific context of developing countries, particularly in the vibrant landscape of South Asia.

## 6. Conclusion

In recent years, online shopping has been one of the fastest-growing trends in Pakistan. However, much research has not been conducted to determine what elements influence Pakistani customers’ behaviour related to online luxury shopping. This study is one of the initial studies to examine the effect of preceding factors in shaping customer attitudes regarding online luxury fashion purchases in emerging economies like Pakistan. The study validates the proposed conceptual framework’s applicability and the usefulness of the technology acceptance model in the luxury market. The research also looks into the interaction effects of risk perceived in online buying, brand awareness, and website atmospherics on attitudes toward luxury products and online luxury purchase intent.

This research not only features the importance but also directs on advancing the research on the intention of consumers to purchase online and it’s determining factors. The results of the correlational analysis indicated that perceived ease of use, perceived usefulness, perceived enjoyment, product knowledge, price consciousness, website atmospherics, and brand awareness positively affected Attitude and online purchase intention. On the other hand, perceived risk has negatively impacted Attitudes and online purchase intention.

Perceived usefulness is not directly associated with online purchase intention, according to the findings of the PLS-SEM; instead, Attitude modulates the relationship between perceived usefulness and online purchase intention. Both directly and indirectly through Attitude, perceived ease of use is associated with online purchase intention. Both directly and indirectly through Attitude, perceived enjoyment is directly linked to online purchase intention. Price consciousness is also directly and indirectly associated with online purchasing intent through Attitude. Although product knowledge is not directly related to online buying intent, Attitude plays a role in moderating the link between product knowledge and online luxury purchase intent.

This particular research is not online in line with the present literature but also provides new insights into the key motivating factors behind the online purchase of luxury fashion products and their association with the Attitude of consumers of emerging markets like Pakistan, thus, adding to the literature.

## 7. Implications

This research comprises fruitful data and results for simultaneous researchers and business professionals. This study addresses the crucial questions in online luxury shopping and answers those questions by accentuating the factors significantly impacting luxury goods consumption among affluent Pakistani online consumers. In addition to highlighting the problems, this research introduces new market strategies to enhance the consumer experience in the online luxury goods market. The consumers are divided into two different yet intertwined categories to develop a suitable method: pleasure-seeking buyers and value-seeking buyers.

The results indicate that perceived enjoyment, price consciousness, and ease of use are all significantly linked with online purchase intention, indicating that perceived ease of use, perceived enjoyment, and price consciousness are positively related to buyers’ attitudes in Pakistan. Companies should introduce different deals and various products to further develop the online luxury market to attract more consumers. As the luxury market is a newly developed concept in Pakistan, the physical luxury stores are few and far between and are only located in the country’s major cities. This produces an enormous potential for the online consumer market far from the major cities. Luxury brands can benefit by introducing online luxury stores to substantially increase their customers, boosting these luxury sectors’ revenue.

There exist many precedences of luxury brands going online and getting benefits from the everlasting impacts of the online market. Luxury brands can follow this precedence and create a luxury collection that targets price-conscious buyers, keeping in check the exclusivity of their products. It might result in the dilution of their brand because of overexposure. As the perceived enjoyment is directly proportional to purchase intention, luxury brands can specifically choose buyers that seek pleasure in buying stuff (pleasure-seeking consumers) by producing an excellent experience in the realm of online shopping and e-commerce stores for the said buyers.

Luxury brands are famous for providing exceptional services to their customers. By applying this mindset to online shops, luxury brands can succeed highly in their online endeavors. To achieve great results, luxury fashion brand managers should create marketing tactics to promote brand recognition to strengthen the brand image and recognition in the minds of consumers, as people tend to buy only brands with which they are familiar. The marketing strategies can also allure more buyers on the Internet, thus significantly enhancing the consumer base on the e-commerce stores. Moreover, luxury brands should create aesthetically pleasing web pages and easier-to-navigate shopping manuals to get more traffic and conversation rate. The results stipulate that web atmospheric positively impacts the Attitude toward purchasing premium fashion goods from online stores, also known as e-commerce stores.

Similarly, the web atmosphere positively affects online premium or luxury purchase intention. To further enhance the experience of prospective online consumers, the creative webpages, high visual appeal, and the provision of advanced tools like augmented reality will not only engage the pleasure-seeking buyers. However, they will also convert aspirational buyers into regular luxury buyers. Lastly, the study confirms that consumers perceive higher risk in online shopping; luxury brands should provide easy exchange and return policies to minimize the risk perceived by online luxury product shoppers. This way, brands can create a list of loyal online customers, further increasing luxury brands’ digital footprint.

## 8. Limitations and future directions

Although this research answers the fundamental questions regarding online luxury shopping and the intent of buyers and consumers, it observes only a few limitations that the researchers can try to minimize by doing further research on the topic. First, the language of the questionnaire used for this research was English. Since English is not the national language of Pakistan, some respondents might have misunderstood a few questions and had marked the wrong answers unknowingly, which is one of the main reasons for the deviation of findings from the stated literature. Secondly, this research comprises the factors that are the hallmark of the Pakistani market and are related to the consumers mainly based in Pakistan. The factors might not be the same for different countries and markets. For instance, the USA has an advanced marketplace and may need additional elements to precisely understand the buyer’s intent regarding online luxury shopping. Thirdly, this research uses the purposive sampling method, which is riddled with limitations. Further studies may utilize probability sampling techniques to procure accurate data and get more generalized results to overcome these limitations.

Additionally, the qualitative study may also produce better results for future research. Fourthly, future studies may use numerous factors such as age, sex, earnings, and other demographic factors to assess the effect of societal elements on online luxury shopping and the behavior of online buyers, in contrast to web atmospherics and the moderating role of perceived risk, that is the basis of this study. Furthermore, future research may use utilitarian and hedonic values as mediator factors instead of Attitude, as done in the current study. Fifthly, future researchers may expand their scope to other luxury sectors such as automobiles, jewelry, etc., or be more specific to a single luxury sector while conducting the study.

## Supporting information

S1 File Consent form. (PDF)

S1 Table Study data. (XLSX)
