# Supplementary material for: Determining online consumer’s luxury purchase intention: The influence of antecedent factors and the moderating role of brand awareness, perceived risk, and web atmospherics
Source: PLoS One. 2024 Feb 23;19(2):e0295514. doi: 10.1371/journal.pone.0295514 (PMC10889853; doi:10.1371/journal.pone.0295514)
Supplement: S1 File — (PDF) [file pone.0295514.s001.pdf]

CONSENT FORM

Dear Respondent,

First of all, I will like to thank all of the people that are focusing on green purchases for their daily use. I am a Assistant professor at the University of the Punjab. I am researching on the "Determining Online Consumer's Luxury Purchase Intention: The Influence of Antecedent Factors and the Moderating Role of Brand Awareness, Perceived Risk, and Web Atmospherics."

Topic of the Research

Determining Online Consumer's Luxury Purchase Intention: The Influence of Antecedent Factors and the Moderating Role of Brand Awareness, Perceived Risk, and Web Atmospherics

Time required for participation

I would encourage you to please take 5 -10 minutes and complete this survey.

Purpose of the research

By conducting this research, the study seeks to enhance the understanding of consumer behavior in the context of online luxury purchases. Specifically, it aims to identify the key factors that influence consumers' intention to purchase luxury products in an online setting. Additionally, the study aims to explore the role of brand awareness, perceived risk, and web atmospherics in shaping consumers' luxury purchase intention..

Confidential information

The accuracy of how you respond to these statements is very crucial for my study. I guarantee that the information which will be gathered from this study, will entirely be used for academic purposes only, will remain strictly confidential, and your identity will stay anonymous.

Voluntary participation

Participation in this study is completely voluntary. If you decide not to participate there will not be any negative consequences. Please be aware that if you decide to participate, you may stop participating at any time and you may decide not to answer any specific question. By signing this form, I am attesting that I have read and understood the information above and I freely give my consent/assent to participate or permission for my employees to participate.

CONSENT FORM

Name: ___________________

Signature: ____

Date: ____

House or Address ___________________

Best Regards

Dr. Hira Aftab

Assistant Professor

Institute of Business & Information Technology

University of the Punjab
